# Factors associated with patterns of plural healthcare utilization among patients taking antiretroviral therapy in rural and urban South Africa: a cross-sectional study

**DOI:** 10.1186/1472-6963-12-182

**Published:** 2012-07-02

**Authors:** Mosa Moshabela, Helen Schneider, Sheetal P Silal, Susan M Cleary

**Affiliations:** 1 Rural AIDS and Development Action Research, School of Public Health, Faculty of Health Sciences, University of Witwatersrand, PO Box 02, Acornhoek, Mpumalanga Province, 1360, Johannesburg, South Africa; 2 School of Public Health, University of the Western Cape, Cape Town, South Africa; 3 Department of Statistical Sciences, University of Cape Town, Cape Town, South Africa; 4 Health Economics Unit, School of Public Health and Family Medicine, University of Cape Town, Cape Town, South Africa

**Keywords:** Antiretroviral treatment, Healthcare utilization, Patient retention, Medical pluralism, Urban–rural, South Africa

## Abstract

**Background:**

In low-resource settings, patients’ use of multiple healthcare sources may complicate chronic care and clinical outcomes as antiretroviral therapy (ART) continues to expand. However, little is known regarding patterns, drivers and consequences of using multiple healthcare sources. We therefore investigated factors associated with patterns of plural healthcare usage among patients taking ART in diverse South African settings.

**Methods:**

A cross-sectional study of patients taking ART was conducted in two rural and two urban sub-districts, involving 13 accredited facilities and 1266 participants selected through systematic random sampling. Structured questionnaires were used in interviews, and participant’s clinic records were reviewed. Data collected included household assets, healthcare access dimensions (availability, affordability and acceptability), healthcare utilization and pluralism, and laboratory-based outcomes. Multiple logistic regression models were fitted to identify predictors of healthcare pluralism and associations with treatment outcomes. Prior ethical approval and informed consent were obtained.

**Results:**

Nineteen percent of respondents reported use of additional healthcare providers over and above their regular ART visits in the prior month. A further 15% of respondents reported additional expenditure on self-care (e.g. special foods). Access to health insurance (Adjusted odds ratio [aOR] 6.15) and disability grants (aOR 1.35) increased plural healthcare use. However, plural healthcare users were more likely to borrow money to finance healthcare (aOR 2.68), and incur catastrophic levels of healthcare expenditure (27%) than non-plural users (7%). Quality of care factors, such as perceived disrespect by staff (aOR 2.07) and lack of privacy (aOR 1.50) increased plural healthcare utilization. Plural healthcare utilization was associated with rural residence (aOR 1.97). Healthcare pluralism was not associated with missed visits or biological outcomes.

**Conclusion:**

Increased plural healthcare utilization, inequitably distributed between rural and urban areas, is largely a function of higher socioeconomic status, better ability to finance healthcare and factors related to poor quality of care in ART clinics. Plural healthcare utilization may be an indication of patients’ dissatisfaction with perceived quality of ART care provided. Healthcare expenditure of a catastrophic nature remained a persistent complication. Plural healthcare utilization did not appear to influence clinical outcomes. However, there were potential negative impacts on the livelihoods of patients and their households.

## Background

In sub-Saharan Africa, the HIV/AIDS epidemic is stabilizing. In 2009, a reduction of 18% in new infections was observed compared to 2001 [1]. However, only 36% of people in need of antiretroviral therapy (ART) were receiving it in 2009 [1]. With an estimated 5.6 million people living with HIV, South Africa’s epidemic remains the largest in the world [1]. According to Adam and Johnson, coverage of ART among those who were in need of ART was only 40% in 2008, although South Africa boasts the largest national ART program in the world [2]. However, increasing concerns about the sustainability of the program are linked to patient’s use of a wide range of health care options, which may pose a threat to patient retention in ART care [3,4].

Patient retention in care is arguably the most important factor determining long-term sustainability of antiretroviral therapy (ART) programs [5]. Giordano et al. demonstrated that retention in care predicts survival among ART patients [6]. However, patient attrition in Sub-Saharan Africa is currently as high as one in three after two years on ART [7-9]. Studies on retention in care are yet to investigate the role of multiple provider usage by ART patients, and how this practice may affect continuity of ART-related care [10]. Indeed patients on ART receive care in the context of plural healthcare systems available to them, and some may combine medical treatment modalities offered elsewhere with ART [2,11,12]. The use of traditional, complementary and alternative healthcare systems has been reported among ART patients in both developed and developing countries [2,11,12]. Plural healthcare usage may complicate the chronic care of ART patients, potentially leading to increases in losses to follow up [10,13].

Variants of plural healthcare utilization such as doctor- or healer-shopping [14,15] and self-treatment [16] have been described among chronically-ill non-HIV/AIDS populations, and are said to vary by geographic location [14]. The extent to which plural healthcare patterns and their determinants exist among ART patients, and affect their healthcare usage, remains unknown [2,10]. In order to inform interventions aimed at patient retention and continuity of care, we used a patient perspective to investigate factors associated with patterns of plural healthcare utilization among ART patients in both urban and rural South Africa. Although health care providers may adequately offer services to the best of their ability, our demand-side approach recognizes that legitimacy of health care and patterns of use are decided upon by patients [17]. In this study, we employed the ‘A- framework’ of access to healthcare [18,19] to examine availability, acceptability and affordability of care. Our findings suggest that push factors within ART clinics and high cost burden on households are associated with increased plural healthcare utilization.

## Methods

### Study design and population

As part of detailed case studies of healthcare access and equity in four sub-districts of South Africa, we conducted cross-sectional studies among ART users (See Table 1). We purposefully selected four sub-districts, two rural and two urban, located in four provinces following consensus between research partners and policy-makers. While such a strategy cannot generate a representative sample, the settings reflect some of the diversity of context typical of South Africa. Table 1 outlines the study sites in more detail, demonstrating that both urban sites and rural sites varied in population size. The two rural sites are also existing demographic surveillance sites, known for high levels of circular labor migration. The urban site in Western Cape and the rural site in Kwa-Zulu Natal are considered to have decentralized ART programs, whereas the other two have highly-centralized ART programs. Since ART is primarily delivered through accredited national public sector roll-out facilities, only users of these programmes were recruited in each of the four settings. A total of 13 ART facilities were selected for this study. In each sub-district, all accredited facilities were included where possible, and where multiple facilities existed, self-weighting stratified or probability proportional to size methods were used to select facilities using data on the total number of users in each facility. A total sample of 1266 users of ART was obtained across the four sites using a systematic random sampling method, ranging from 300 to 331 in each sub-district. Only adult ART patients, 18 years and older, with a minimum of two weeks since ART initiation were included in this study, and their ART clinic records were reviewed for biological markers. The sampling procedure aimed to ensure a representative sample in each of the four sites. Following selection of facilities, ART clinic appointment and attendance registers were used to estimate the total number of patients scheduled to attend the ART clinic and to select a random position in the queue of patients on the day of data collection. The fixed interval (*n*th position) in the queue was chosen depending on the size of the facility and the total numbers expected on the day of data collection. The selection process was also spread out through the entire day in order to capture patients presenting at different times of the day. In each site, once data collection commenced, it was continued on every ART clinic day until the desired sample had been achieved. The data collection occurred between April 2008 and March 2009.

**Table 1:** Study sites and sampling methods presented under elected provinces and sub-districts

**Province**	**Western Cape**	**Gauteng**	**Mpumalanga**	**Kwa-Zulu Natal**
Site (Sub-district) selected	Mitchell’s Plain	Soweto	Bushbuckridge	Hlabisa
Classification	Urban	Urban	Rural	Rural
Population	290000	1100000	620000	228000
ART facilities	03	07	02	16
Facility sampling method	All	Self-weighting Stratified	All	Probability Proportional to Size
Facilities sampled	03	03	02	05
Participants sampled	323	331	312	300
Participants sampling method	Systematic	Systematic	Systematic	Systematic

### Data collection

Structured questionnaires were used to collect data during exit interviews (see Additional file 1). Data were collected on socio-demographic factors, availability and affordability of ART services, psychosocial support for ART, acceptability and quality of ART care, utilization and clinical outcomes, and household assets were used to estimate socioeconomic status (SES). Participants were interviewed on the day of their clinic visit to the ART service point. Trained research assistants conducted face-to-face exit interviews in XiTsonga, Afrikaans, IsiXhosa, IsiZulu, Sepedi or English depending on the participant’s preference. Exit interviews were linked by unique identifiers to an HIV clinic record data extraction form that obtained additional clinical data such as CD4 count and viral load levels on the same day as the interview.

Participants were asked two key questions in relation to additional health care sought in the month prior to their clinic visit. The first question was: “Apart from visits to this clinic for your ARVs, have you used this clinic or any other health service in the last four weeks? If so, how many visits did you have, and how much did you have to pay the provider for each?” Participants were prompted with a series of choices, including the ART clinic itself (not for ARVs), public primary healthcare (PHC) clinic or hospital, a private chemist, doctor or hospital, and Tuberculosis (TB) or antenatal (ANC) clinics. The second question was “Have you spent any other money on health care in the past month (e.g. traditional medicines, spaza shops, special food, etc.)? If so, how much have you spent?” The first question relates to the concurrent use of additional providers, and represents plurality of health service and provider usage, and the second question to purchase of health care products and substances, representing a form of self-care behavior.

### Data reduction and analysis

Data were double-entered into EpiData v3.1 and imported into STATA v10 (Statacorp, College Station, Texas) for analysis. Descriptive statistics of plural healthcare patterns and constructs, stratified by urban and rural residence, were generated and bivariate analysis conducted with the chi-squared test and logistic regression. Multiple logistic regression analyses were used to identify predictors of utilization patterns, and we present adjusted odds ratios and 95% confidence intervals. The factors assessed included a socio-economic index, access barriers (including perceptions of quality) and biological outcomes such as baseline and follow-up CD4 counts and viral load suppression. An index for SES was constructed using multiple correspondence analysis, described elsewhere [20]. Catastrophic healthcare expenditure was computed as mean monthly healthcare-related expenditure of more than 15% of mean monthly household expenditure. ART-related knowledge was generated using a combination of three questions: knowledge of recent CD4 count, and correct responses to two questions, do you stop ART when weight improves, and does ART cure HIV/AIDS.

Provider pluralism represents use of any additional provider, irrespective of additional self-help practices. Self-care pluralism refers to purchase of any product or substance for health care purposes, and excluding additional provider utilization. A third dependent variable was generated by a combination of plural provider and self-care variables, referred to as plural healthcare utilization. Plural healthcare is used in particular way in this study, whereby use of physicians and private chemists is measured alongside traditional healers as a reflection all healthcare service modalities available in this context. Multivariate logistic models were fitted for provider pluralism, self-care pluralism and the combined variable of plural healthcare utilization. Participants who sought additional care from their regular ART clinic were excluded from provider pluralism. For purposes of analysis, TB and ANC users were included in the initial description of plural healthcare patterns, but excluded in the models for determinants and implications, as there are clearly defined indications for use of TB and ANC services.

### Ethical considerations

Ethical approval was obtained from Universities of Witwatersrand, Kwa-Zulu Natal, Cape Town and the respective Provincial Health Research Committees. Written informed consent was obtained separately for the interview, and for the HIV clinic record review.

## Results

### Characteristics of study participants

As shown in Table 2, participants were distributed approximately equally between urban (52%) and rural (48%) sites, and were predominantly Black African (98%). In keeping with the gender distribution of most ART clinics, 74% of participants were female. Most participants were aged below 40 years (61%), never married (55%) and unemployed (78%). While the majority of participants did not possess medical aid insurance (98%), 42% were enrolled on the temporary disability grant intended for financing of healthcare needs. The SES measure allowed for equally-sized groupings across all quintiles (20%), indicating lack of clumping or truncation [21]. At initiation of ART, 78% of participants had a baseline CD4 count above 50 cells/ul.

**Table 2:** Characteristics of ART-using participants in relation to plural healthcare usage

**Variable**	**Category**	**N**	**%**	**Non-Plural (N, %)**		**Plural (N, %)**		**P-value**
Total	All	1266	100	838	66	428	34	
Sex	Female	933	74	625	75	308	72	
	Male	333	26	213	25	120	28	0.317
Age	18–29	252	20	171	21	81	19	
	30–39	515	41	351	42	164	38	
	40–49	337	27	210	25	127	30	
	50 and above	158	12	103	12	55	13	0.319
Race	African	1236	98	819	98	417	98	
	Coloured	28	02	19	02	9	02	0.860
Marital Status	Married	250	20	163	19	87	20	
	Living with Partner	91	7	49	6	42	10	
	Divorced or Separated	113	9	90	11	23	6	
	Widowed	112	9	73	9	39	9	
	Never Married	699	55	463	55	236	55	0.007
Education	None	126	10	87	11	39	09	
	Primary	313	25	194	23	119	28	
	Secondary	573	45	388	46	185	43	
	Matric and above	252	20	167	20	85	20	0.317
Employment	None	981	78	639	76	342	80	
	Part-time	124	10	80	10	44	10	
	Full-time	158	12	116	14	42	10	0.115
Residence	Rural	612	48	351	42	261	61	
	Urban	654	52	487	58	167	39	<0.001
Disability Grant	No	733	58	515	62	218	51	
	Yes	532	42	322	38	210	49	<0.001
Medical Aid	No	1242	98	829	99	413	96	
	Yes	22	02	07	01	15	04	0.001
Socio-economic Status	Quintile 1 (Poorest)	253	20	142	17	111	26	
	Quintile 2	253	20	165	20	88	21	
	Quintile 3	253	20	167	20	86	20	
	Quintile 4	553	20	189	22	64	15	
	Quintile 5 (Richest)	254	20	175	21	79	18	<0.001
Baseline CD4 count	Above 50 cells	904	78	591	77	313	79	
	Below 50 cells	257	22	172	23	85	21	0.644

### Patterns of plural healthcare utilization post-HAART initiation

**Figure 1:**
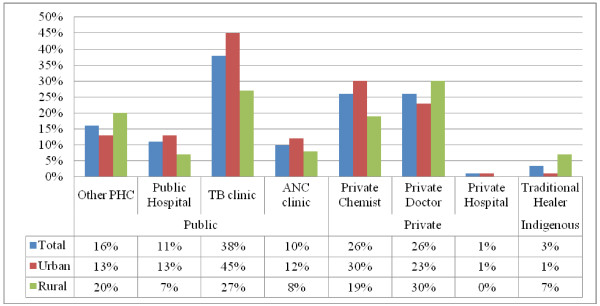
Patterns of using different types of additional providers demonstrate higher public hospital (p = 0.126), private hospital (p = 0.408), private chemist (p = 0.023), TB (p = 0.001) and ANC (p = 0.323) in urban areas, whereas public PHC (p = 0.103), private doctor (p = 0.149) and traditional healers use (p = 0.005) were higher in rural areas.

Just under one-fifth (18.9%) of participants visited additional healthcare providers over and above their regular ART clinical care. As shown in Figure 1, provider pluralists visited chemists, doctors and hospitals in the private sector, primary healthcare (PHC) clinics and hospitals in the public sector, and traditional healers in the indigenous sector. A further 14.9% of participants purchased products or substances for purposes of healthcare, which we refer to as self-care pluralism. The two variants of plural healthcare pluralism, provider and self-care, occurred in a combined 33.8% of all respondents.

The types of provider used varied between urban and rural sites (Figure 1). Urban residents made greater use of private chemists (30% vs. 19%), while rural areas were more likely to use traditional healers (7% vs. 1%). Usage of private doctors was slightly higher among rural residents (30% vs. 23%). A slightly greater proportion of urban residents used public hospitals (13% vs. 7%), whereas a greater proportion of rural residents used PHC facilities (20% vs. 13%) relative to urban residents. Traditional healers were used more by rural residents than their urban counterparts (7% vs. 1%). Within a period of one month, an average single visit to an additional provider was reported by the participants. Mean and range of visits were identified as follows among plural provider users: private chemists (01, 01–03), private doctors (01, 01–06), private hospitals (01, 01–01), PHC clinics (01, 01–03), public hospitals (02, 01–10) and traditional healers (01, 01–03).

### Additional expenditure by plural healthcare users

**Figure 2:**
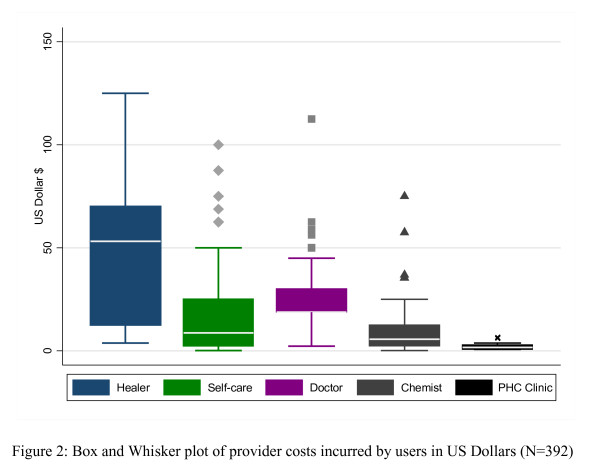
**Box and Whisker plot of costs of use of additional providers in the prior 4 weeks (US$).** Costs incurred among plural health care users demonstrate exorbitant amounts associated with the use of traditional healers as opposed to low costs spent when using primary health care clinics. The costs for self-care, private doctors and private chemists were also considerable.

Participants incurred expenses related to both provider and self-care pluralism, measured by reported direct healthcare costs only, as shown in Figure 2. Although utilization of traditional healers (N = 11) was uncommon, the highest amount of expenses reported (USD125) was paid to these providers. Private chemists (Inter-quartile range [IQR] USD3-USD13, N = 86) and doctors (IQR USD19-USD30, N = 88) were also relatively costly for participants, with median costs of USD6 and USD19 respectively. The costs of self-care practices (N = 154) were also high, with a median of USD9 (IQR USD3-USD25). However, usage of PHC clinics (IQR USD1-USD3, N = 53) was less costly with a maximum amount of USD7 spent.

Despite the likelihood that some of the indirect and direct costs may not have been fully captured, and for a largely unemployed source population, the patients surveyed incurred large costs to finance their healthcare. Nearly one-fifth (18.7%) of the total sample reported having to borrow money (18.7%) and at times sell their belongings (4.9%) in order to finance healthcare. Borrowing was more common among plural care users (P < 0.001), and rural residents were 6.8 times more likely to borrow than urban residents (Adjusted Odds Ratio [aOR] 6.75, 95% Confidence Interval [95%CI]: 4.73–9.64). Amongst those borrowing money, plural care users (63.0%) were more likely to report difficulty incurring the costs of healthcare use than non-plural users (37.0%) (P = 0.006).

When the degree of catastrophic healthcare expenditure was measured, 34.8% of plural healthcare users showed catastrophic levels as opposed to 6.9% among patients not using additional healthcare (P < 0.001). Catastrophic level of healthcare expenditure was identified in 77.2% of plural healthcare users with rural origin compared to 22.8% of urban residents (P < 0.001). Using the socioeconomic distribution of participants by quintiles ranging from poorest to richest shown in Table 3, urban plural care users were found to be of a higher SES in comparison to their rural counterparts (P < 0.001).

**Table 3:** Rural and urban distribution of socio-economic status among plural healthcare users (N = 428)

	**Rural**	**Urban**
1. Poorest	38%	7%
2.	30%	6%
3. Middle	20%	20%
4.	9%	24%
5. Richest	3%	43%

### Factors associated with plural healthcare utilization

The use of plural healthcare was higher in rural participants (aOR 1.97, 95%CI: 1.12–3.44). As shown in Table 4, higher rates of pluralism occurred among those who possessed medical aid insurance (aOR 6.15, 95%CI: 2.31–16.33). Having a disability grant was associated with 35% increased odds of plural healthcare. Although SES was not statistically significant when adjusted for other factors, creating debts by borrowing money (aOR 2.68, 95%CI: 1.87–3.84) to finance healthcare were strongly associated with plural healthcare usage. Borrowing money was also associated with catastrophic household expenditure (aOR 3.26, 95%CI: 2.25–4.73).

**Table 4:** Predictors of healthcare pluralism among ART patients

**Variables**	**Simple logistic regression**			**Multiple logistic regression**		
	**Odds ratio**	**95% Confidence interval**	**P-value**	**Odds ratio**	**95% Confidence interval**	**P-value**
Age (Younger <30 years)	0.91	0.68–1.22	0.526	-	-	-
Marital status (Divorced)	0.47	0.29–0.76	0.001	0.64	0.38–1.07	0.091
Education (None)	0.86	0.58–1.28	0.468	-	-	-
Employment (None)	1.22	0.91–1.62	0.179	-	-	-
Residence (Rural)	2.17	1.71–2.75	<0.001	1.97	1.12–3.44	0.018
Medical aid (Yes)	4.30	1.74–10.63	0.002	6.15	2.31–16.33	<0.001
Disability Grant (Yes)	1.54	1.22–1.95	<0.001	1.35	1.02–1.78	0.037
Socioeconomic status (Poor)	1.50	1.17–1.90	0.001	-	-	-
Borrowing (yes)	3.84	2.86–5.14	<0.001	2.68	1.87–3.84	<0.001
Difficulty incurring costs (yes)	1.49	1.11–2.01	0.008	-	-	-
Sell Property for health care (Yes)	5.08	2.40–10.72	<0.001	2.26	0.99–5.20	0.054
Closest ART facility (Yes)	1.49	1.05–2.10	0.025	-	-	-
Collection of ART (Monthly)	2.13	1.53–2.96	<0.001	-	-	-
Treatment supporter (Yes)	2.05	1.57–2.67	<0.001	1.44	1.06–1.97	0.021
Home visit by health worker (Yes)	1.31	1.02–1.68	0.032	1.38	0.99–1.94	0.061
Provider preference (Nurse)	1.42	1.11–1.83	0.006	-	-	-
Ever left without help (Yes)	1.95	1.14–3.32	0.014	-	-	-
Queue (Too long)	0.80	0.63–1.02	0.073	-	-	-
Privacy in consultation (No)	1.54	1.19–1.98	0.001	1.50	1.08–2.08	0.015
Language barrier (Yes)	0.35	0.23–0.53	<0.001	0.57	0.35–0.91	0.019
Staff disrespect (Agree)	2.46	1.94–3.13	<0.001	2.07	1.54–2.79	<0.001
Dirty facilities (Agree)	1.09	0.81–1.47	0.558	-	-	-
ART missed doses (Ever)	1.51	1.06–2.17	0.024	-	-	-
ART knowledge (Low)	1.21	0.94–1.54	0.133	-	-	-
Missed clinic visit (Yes)	1.00	0.60–1.68	0.999	-	-	-

Log Likelihood −656, Pseudo R^2^ 15%, Goodness-of-fit p-value 0.25.

The presence of social support in the form of treatment buddies increased the odds of plural healthcare by 44%. Finally, several variables related to the quality of care experienced by participants in the ART clinic showed association with use of plural healthcare. Perceived lack of privacy in the ART consultation rooms (aOR 1.50, 95%CI: 1.08–2.08) and disrespect by ART providers (aOR 2.07, 95%CI: 1.54–2.79) was associated with plural healthcare use, whereas participants who encountered language barriers with ART providers had 43% reduced usage of plural healthcare as opposed to those who did not.

### Variants of plural healthcare utilization: associated factors

We constructed separate regression models for the two variants of plural healthcare utilization, namely, provider pluralism and self-care pluralism, and the results are presented below as well as in Table 5.

**Table 5:** Predictors of provider and self-care pluralism patterns among ART patients

**Variables**	**Provider simple logistic regression**			**Provider multiple logistic regression**			**Self-care simple logistic regression**			**Self-care multiple logistic regression**		
	**Odds ratio**	**95% Confidence interval**	**P-value**	**Odds ratio**	**95% Confidence interval**	**P-value**	**Odds ratio**	**95% Confidence interval**	**P-value**	**Odds ratio**	**95% Confidence interval**	**P-value**
Age (Younger <30 years)	1.19	0.84–1.67	0.325	-	-	-	0.66	0.43–1.01	0.056	-	-	-
Marital status (Divorced)	1.11	0.84–1.48	0.457	-	-	-	0.87	0.64–1.19	0.383	0.43	0.16–1.13	0.087
Education (None)	0.64	0.38–1.10	0.104	-	-	-	1.23	0.75–2.00	0.406	-	-	-
Employment (None)	0.92	0.66–1.29	0.639	-	-	-	1.62	1.07–2.45	0.023	-	-	-
Residence (Rural)	0.86	0.64–1.13	0.279	-	-	-	5.72	3.90–8.39	<0.001	5.91	1.90–18.40	0.002
Disability Grant (Yes)	-	-	-	-	-	-	2.59	1.88–3.56	<0.001	1.93	1.33–2.80	0.001
Medical Aid (Yes)	7.91	3.28–19.08	<0.001	7.59	3.06–18.78	<0.001	-	-	-	-	-	-
Socioeconomic status (Poor)	0.71	0.53–0.95	0.023	0.67	0.45–0.98	0.037	3.03	2.20–4.18	<0.001	-	-	-
Borrowing (yes)	1.76	1.27–2.46	0.001	1.90	1.29–2.81	<0.001	4.08	2.92–5.71	<0.001	2.42	1.62–3.61	<0.001
Difficulty incurring costs (yes)	0.77	0.54–1.10	0.151	-	-	-	0.66	0.44–0.99	0.050	-	-	-
Collection of ART (Monthly)	1.26	0.87–1.82	0.230	-	-	-	3.76	2.10–6.72	<0.001	-	-	-
Treatment supporter (Yes)	1.49	1.08–2.04	0.014	-	-	-	2.31	1.58–3.40	<0.001	1.58	1.00–2.51	0.050
Support group (Yes)	1.15	0.86–1.56	0.343	-	-	-	0.69	0.49–0.99	0.043	-	-	-
Having left without help (Yes)	2.87	1.65–4.99	<0.001	2.67	1.43–4.99	0.002	0.66	0.28–1.56	0.342	-	-	-
Queue (Too long)	1.22	0.90–1.65	0.205	-	-	-	0.55	0.40–0.75	<0.001	-	-	-
Provider preference (Nurse)	1.04	0.77–1.40	0.814	-	-	-	1.86	1.30–2.66	0.001	0.52	0.31–0.89	0.017
Non-adherence (Able to disclose)	1.21	0.83–1.78	0.322	-	-	-	0.49	0.29–0.84	0.010	-	-	-
Privacy in consultation (No)	0.98	0.73–1.32	0.884	-	-	-	2.39	1.63–3.51	<0.001	1.83	1.13–2.97	0.014
Language barrier (Agree)	0.52	0.32–0.84	0.007	0.55	0.32–0.96	0.034	0.32	0.17–0.63	0.001	-	-	-
Staff disrespect (Agree)	1.46	1.10–1.95	0.008	1.43	1.02–2.00	0.036	3.03	2.20–4.17	<0.001	1.92	1.30–2.85	0.001
Dirty facilities (Agree)	1.53	1.10–2.14	0.013	1.40	0.97–2.03	0.074	0.64	0.41–0.99	0.049	0.57	0.34–0.96	0.033
ART missed doses (Ever)	1.00	0.67–1.51	0.990	-	-	-	2.40	1.33–4.32	0.004	-	-	-
ART knowledge (Low)	0.79	0.58–1.08	0.142	-	-	-	1.77	1.30–2.43	<0.001	-	-	-
Missed ART clinic visit (Yes)	1.58	0.91–2.78	0.104	-	-	-	0.43	0.17–1.10	0.079	-	-	-

Log Likelihood −547, Pseudo R^2^ 07%, Goodness-of-fit p-value 0.23 (Provider), Log Likelihood −384, Pseudo R^2^ 25%, Goodness-of-fit p-value 0.96 (Self-care).

### Provider pluralism

Lower SES was a predictor of reduced provider pluralism (aOR 0.67, 95%CI: 0.45–0.98). Ability to finance health care, such as seen among those who used medical aid insurance (aOR 7.59, 95%CI: 3.06–18.78), was predictive of provider pluralism. However, debt creation was higher among provider pluralists by 90% (Table 5).

xParticipants who on any previous occasion had to leave the ART clinic without receiving help were 2.7 times more likely to use additional providers. In addition, perceptions of ART provider disrespect showed 43% increased odds of provider pluralism. However, experiences of language barriers (aOR 0.55, 95%CI: 0.32–0.96) were associated with reduction in provider pluralism. Therefore, factors representing poor quality of care were predictive of provider pluralism.

### Self-care pluralism

Rural-dwelling (aOR 5.91, 95%CI: 1.90–18.40) and temporary disability grant enrolment (aOR 1.93, 95%CI: 1.33–2.80) were found to be associated with self-care pluralism. The self-care category was 2.4 times more likely to borrow money in order to finance healthcare. Those engaging in self-care were 58% more likely to have a treatment supporter, a form of social support, although the level of statistical significance was marginal (Table 5).

Self-care utilization was associated with 83% and 92% increased odds in lack of privacy during consultation and perceived disrespect for patients by clinic staff, respectively. Participants who preferred to be seen by a nurse provider (aOR 0.52, 95%CI: 0.31–0.89) on their regular clinic visit were less likely to engage in self-care pluralism, as opposed to those who preferred a doctor. Surprisingly, participants who perceived facilities to be dirty were also less likely to engage in self-care pluralism (aOR 0.57, 95%CI: 0.34–0.96). However, dirty facilities predicted plural provider utilization in bivariate logistic regression (OR 1.53, 95%CI: 1.10–2.14).

### Clinical factors

CD4 count level at ART initiation was not associated with plural healthcare (aOR 0.93, 95%CI: 0.70–1.25), adjusted for age, sex, education, SES, closeness to ART facility and ART knowledge. Among participants with recorded recent CD4 count results (59%), improvement in CD4 results was not associated with plural healthcare utilization (aOR 1.06, 95%CI: 0.72–1.57). When viral loads were retrievable (66%), viral suppression (70%) was not associated with plural use of healthcare (aOR 0.97, 95%CI: 0.83–1.58). However, in bivariate but not multivariate logistic regression models, self-care pluralism was associated with poor levels of ART-related knowledge (OR 1.77, 95%CI: 1.30–2.43) and missed doses of ART (OR 2.40, 95%CI: 1.33–4.32), but not with missed ART clinic visits (OR 0.43, 95%CI: 0.17–1.10). Some patients (8.0%) had previously received ART from facilities other than their regular clinic, a practice associated with provider pluralism (OR 1.76, 95%CI: 1.01–3.09).

## Discussion

In light of growing interest in ART-related healthcare pluralism, this multisite South African study examined utilization of additional health providers and self-purchased health products concurrently with ART services. The study provides insights into factors associated with these plural healthcare practices among ART patients, and how these factors differ between urban and rural settings. The results suggest increased plural healthcare utilization, inequitably distributed between rural and urban areas, is largely a function of higher SES, better ability to finance healthcare and factors related to poor quality of care in ART clinics. Healthcare expenditure of a catastrophic nature to households remained a persistent consequence associated with plural healthcare utilization. Notably, plural healthcare utilization was neither associated with biological markers of ART success, CD4 count and viral load, nor scheduled visits to the ART clinic.

Provider-related (19%) and self-care (15%) pluralism are conceptually distinct variants in that the former is driven by usage of healthcare providers, and the latter by self-help behavior. However, they both represent the same phenomenon of seeking complementary healthcare to ART. A study by Rosen et al. found self-care to be as high as 60% among HIV/AIDS patients, while 12% paid for other medical care in the preceding week [22]. In our study, provider and self-care pluralism were more common in urban and rural settings respectively, suggesting possible geographic inequities. Horstmann et al*.* hypothesized that differences in multiple service usage by ART patients between urban and rural settings were much more likely to occur due to inequitable distribution of health providers and resources [10]. Our study found provider pluralism patterns to involve public, private and indigenous sectors, and this concurs with previous qualitative research [2]. The use of self-care practices and traditional healers were more common in rural areas, whereas the private sector was used largely by urban residents, who also had a much higher SES. Several household studies have identified SES as an important determinant of using or choosing health providers [23-25].

Some similarities were identified between provider and self-care pluralism, notwithstanding geographic differences. The direct costs of healthcare were higher among users of traditional healers who were mostly rural patients. Although these costs were high, alternatives to cash payments through payment in kind or on credit renders traditional healthcare affordable [26]. Urban patients spent money largely on private chemists and doctors. Plural healthcare users, both provider-related and self-care, created debts by raising money to finance healthcare, a known practice via social networks [26]. In addition, provider pluralism and self-care practices were associated with possession of medical aid insurance and temporary disability grants respectively, both of which may increase the ability to finance healthcare utilization. Furthermore, geographic disparities in SES may explain high levels of catastrophic household expenditure in rural areas, which may in turn account for the increased need to borrow money so as to finance healthcare. Previous studies have shown that catastrophic expenditure associated with chronic care, as is the case with ART, may result in depletion of household livelihoods with a greater effect among rural residents [23,25,27]. Most likely, the higher SES among urban ART users may have provided some resilience against catastrophic household expenditure.

Further implications of provider and self-care pluralism pertain to the direct role of the ART services. Both provider and self-care pluralism increased when patients experienced disrespect by the healthcare team in the ART clinic. A study by Magnus et al. showed that perceived respect at the ART clinic was associated with increased patient retention, a result of perceived good quality healthcare [28]. On the contrary, perceived poor quality of care was identified in other studies as an important reason for poor patient retention or attendance in the ART clinic [29,30]. Other poor quality of care factors identified in this study included lack of privacy during consultations and having to leave the ART clinic without receiving help. These factors may act as barriers to care, and recourse to different medical systems is known to reduce barriers to HIV care in certain cases [31]. Furthermore, the role of treatment supporters in increasing plural healthcare utilization is worth noting, and this form of social support is also necessary to improve adherence to ART [30]. Social influence, manifesting in the context of cultural networks, may function against the goals of ART services particularly when plural healthcare is discouraged in the ART clinic [32,33]. However, a collaborative beneficial effect may be seen in a coordinated plural healthcare system [33].

With regards to healthcare outcomes, only self-care pluralism was associated with low knowledge regarding ART care and reports of missed treatment doses in the preceding 6 months. Poor ART-related knowledge was associated with low level of education in a study by Nachega et al. [34], while in this study it was associated with the rural context and low SES associated with self-care behavior. However, clinic visits in the preceding 6 months and immunological and virological markers were neither affected by provider nor self-care pluralism. Limiting the study were the low levels of recorded biological markers. Future research needs to explore these outcomes in the context of complete results. Furthermore, this study could not establish the direction of cause and effect due to a cross-sectional design, and longitudinal studies are needed to better describe determinants and consequences of plural healthcare utilization. In addition, studies are needed to establish the clinical, personal and contextual appropriateness of plural healthcare utilization, if effective integrated healthcare interventions are to be designed. Furthermore, plural health care utilization is a complex concept that is used in particular way in this study and challenging to measure by quantitative tools. Some benefits of using a hybrid of health care modalities may have included non-medical or psychological relief not captured by economic or access variables used in this study.

## Conclusion

In conclusion, increased plural health care utilization, irrespective of disparities between urban and rural contexts, may be an indication of patients’ dissatisfaction with the perceived quality of ART care provided. The different patterns are driven by socio-economic status, ability to finance health care and social influence. Notably, plural health care usage in ART care may not carry immediate implications for clinical outcomes. Further longitudinal research is needed to investigate reliably the impact of plural healthcare utilization on clinical and public health outcomes. However, the identified catastrophic financial consequence of plural health care usage carries implications for livelihoods of ART patients and their households, and therefore warrants urgent interventions. In spite of the cost burden incurred, patients continue to seek additional healthcare while on ART, a crude measure of the importance attached to the sought additional healthcare. Appropriate interventions targeted at covariates of plural care practices may serve to improve health system responsiveness and patient satisfaction with ART care, and help alleviate the financial strain imposed on HIV/AIDS-affected households by plural health care usage. The result may be an increase in patient retention and improved continuity of care for ART patients.

## Abbreviations

HIV: Human immune-deficiency virus; AIDS: Acquired immune-deficiency syndrome; ART: Antiretroviral therapy; ARVs: Antiretroviral therapy; SES: Socioeconomic status; aOR: Adjusted odds ratio; 95%CI: 95% confidence interval; OR: Odds ratio; p: P-value; USD: United States Dollars; IQR: Inter-quartile range; PHC: Primary healthcare; TB: Tuberculosis; ANC: Antenatal care.

## Competing interests

The authors declare that they have no competing interests.

## Authors’ contributions

MM participated in conception and design, collection of data, performed the statistical analysis and drafted the manuscript. HS participated in conception and design, interpretation of data and critical revision of the manuscript. SPS assisted with the statistical analysis and interpretation of data, and critically reviewed the manuscript. SMC assisted with study design, accumulation of data, interpretation of health economics concepts, and critically revised the manuscript. All authors read and approved the manuscript.

## Authors’ information

MM (MBChB, MMed, Dip HIV Man, PhD) is a senior lecturer and director of Rural AIDS and Development Action Research within the School of Public Health at the University of Witwatersrand, Johannesburg. HS (MBChB, MMed) is a Professor at the School of Public Health, University of the Western Cape, and principal investigator of the Researching Equitable Access to Health Care (REACH) project. SPS (MSc) is statistician working on the REACH project. SMC (PhD) is an Associate Professor and director of the Health Economics Unit at the University of Cape Town.

## Pre-publication history

The pre-publication history for this paper can be accessed here:

http://www.biomedcentral.com/1472-6963/12/182/prepub

## Supplementary Material

Additional file 1 Patient exit interview questionnaire. Click here for file
