# Supplementary material for: Factors associated with patterns of plural healthcare utilization among patients taking antiretroviral therapy in rural and urban South Africa: a cross-sectional study
Source: BMC Health Serv Res. 2012 Jul 2;12:182. doi: 10.1186/1472-6963-12-182 (PMC3465179; doi:10.1186/1472-6963-12-182)
Supplement: Additional file 1 — Patient exit interview questionnaire. [file 1472-6963-12-182-S1.doc]

| **REACH** **PATIENT EXIT INTERVIEW QUESTIONNAIRE****tracer: ART** | |
| --- | --- |
| 0.1  Date of interview | dd mm yyyy |
| 0.2  Interviewer  name | _____________________________________________________________ |
| 0.3  Patient number | **Place sticker here** |
| 0.4  Start time of interview | _____________:________________  hour min |
| 0.5  Site (name of facility) | ________________________________________ |

**Instructions for interviewers:**

Questions or parts of questions that do not always need to be read out and instructions are **in highlighted text**.

Skips indicating which questions can be left out are indicated by arrows

**Go to**

Unless specifically asked to do so, options do not need to be read out.

| **SECTION 1: SOCIOECONOMIC AND DEMOGRAPHIC BACKGROUND QUESTIONS ABOUT THE RESPONDENT, HIS/HER HOUSEHOLD AND HOUSEHOLD HEAD** | | | | | | | |
| --- | --- | --- | --- | --- | --- | --- | --- |
| **READ OUT**  I am going to start by asking you a few questions about you and your household. When I talk about your household, I am including all the people who live in your house and who share the same food with you.  When I talk about your household head, this is the person who usually makes the important decisions in the household. | | | | | | | |
| **1.1**  Sex | | | | | Male | | 1 |
|  |  |  |  |  | Female | | 2 |
| **1.2**  Note the race of the respondent. If you are not certain, ask: How would you describe yourself racially? | | | | African/Black | | | 1 |
|  |  |  |  | Coloured | | | 2 |
|  |  |  |  | Asian/Indian | | | 3 |
|  |  |  |  | White | | | 4 |
|  |  |  |  | Other (specify) | | | |
| **1.3**  What was your age at your last birthday?  Fill in one block only | ____________________  Year born | | | | ____________________  Years | | |
| **1.4**  Who is the head of your household? By this, I mean, who is the person who usually makes the important decisions in the household. Indicate relationship e.g. father, mother not name. | | | ___________________________________  Relationship | | | | |
| **1.5**  Code sex of HHH. If not clear ask: What is the sex of your HHH? | | Male | | | | | 1 |
|  |  | Female | | | | | 2 |
| **1.6**  Code position in HH of respondent. If unclear, ask:    What is your position in the household, in relation to the household head such as …read out a few relevant options.  Tick one block only | | Head/acting head | | | | | 1 |
|  |  | Husband/wife/partner | | | | | 2 |
|  |  | Son/daughter/stepchild/adopted child | | | | | 3 |
|  |  | Brother/sister/step brother/step sister | | | | | 4 |
|  |  | Father/mother/step father/step mother | | | | | 5 |
|  |  | Grandparent/great grandparent | | | | | 6 |
|  |  | Grandchild/great grandchild | | | | | 7 |
|  |  | Other relative (e.g. in laws or aunt/uncle) | | | | | 8 |
|  |  | Non-related persons (tenant, boarder, lodger) | | | | | 9 |
|  |  | Don’t know | | | | | 99 |
|  |  | Other (specify) | | | | | |
| **1.7**  What was the age of your HHH i.e. husband / father / mother etc**.** at his/her last birthday? fill in one block only | | ____________________  Year born | | | | ____________________  Years | |
| **1.8**  Does your HHH i.e. husband / father / mother etc**.** stay with you for at least 2 weeks each month? | | | | | | Yes | 1 |
|  |  |  |  |  |  | No | 0 |

| **1.9**  What is your current marital status?  Tick one block only | | | | Married | | | | | | | 1 | | |
| --- | --- | --- | --- | --- | --- | --- | --- | --- | --- | --- | --- | --- | --- |
|  |  |  |  | Living with partner | | | | | | | 2 | | |
|  |  |  |  | Widow/widower | | | | | | | 3 | | |
|  |  |  |  | Divorced or separated | | | | | | | 4 | | |
|  |  |  |  | Never married (single) | | | | | | | 5 | | |
|  |  |  |  | Other (specify) | | | | | | | | | |
| **1.10**  What is YOUR highest level of education?  Tick one block only  If the person is NOT the HHH ask  What is the highest level of education of your HHH i.e. husband / father / mother etc. | **Type of education** | | | | | | **You** | | | **Your HHH** | | | |
|  | No schooling | | | | | | 0 | | | 0 | | | |
|  | Highest grade passed in school (1-12) | | | | | |  | | |  | | | |
|  | Completed diploma/certificate | | | | | | 13 | | | 13 | | | |
|  | Completed degree | | | | | | 14 | | | 14 | | | |
|  | Other (specify) | **You** | | | | **Your HHH** | | | | | | | |
| **1.11**  Are you currently employed working or earning money?  If the person is NOT the HHH ask  Is your HHH i.e. husband / father / mother etc. currently employed? | | | | | **Type of employment** | | **You** | | | **Your HHH**  **If no or don’t know go to**  **1.13** | | | |
|  |  |  |  |  | Yes, full-time | | 1 | | | 1 | | | |
|  |  |  |  |  | Yes, part-time | | 2 | | | 2 | | | |
|  |  |  |  |  | No | | 3 | | | 3 | | | |
|  |  |  |  |  | Don’t know | | 99 | | | 99 | | | |
| **1.12**  If respondent employed ask:  Are you self-employed or do you work for someone else?  If HHH employed, ask:  Is your HHH i.e. husband / father / mother etc. self-employed or does HE/SHE work for someone else? | | | | | **Type of employment** | | **You** | | | **Your HHH**  **Go to**  **1.14** | | | |
|  |  |  |  |  | Self-employed | | 1 | | | 1 | | | |
|  |  |  |  |  | Employee | | 2 | | | 2 | | | |
|  |  |  |  |  | Don’t know | | 99 | | | 99 | | | |
| **1.13**  If respondent not employed ask:  What are the reasons that you are not employed?  Tick all that apply “Yes” and others “No” | | | **Reason** | | | | | | Yes | | | | No |
|  |  |  | Looking for work | | | | | | 1 | | | | 0 |
|  |  |  | Retired or pensioner | | | | | | 1 | | | | 0 |
|  |  |  | Sick or injured | | | | | | 1 | | | | 0 |
|  |  |  | Pregnant or caring for own children | | | | | | 1 | | | | 0 |
|  |  |  | Caring for other children | | | | | | 1 | | | | 0 |
|  |  |  | Caring for sick/injured | | | | | | 1 | | | | 0 |
|  |  |  | Retrenched | | | | | | 1 | | | | 0 |
|  |  |  | Nothing | | | | | | 1 | | | | 0 |
|  |  |  | Don’t know | | | | | | | | | 99 | |
|  |  |  | Other (specify) | | | | | | | | | | |
| **1.14**  How many adults (18 years or older) live in your household? When I talk about your household, I am including all the people who live in your house and who share the same food with you. | | | | | | | |  | | | | | |
| **1.15**  How many children younger than 18 years live in your household? | | | | | | | |  | | | | | |

| **1.16**  Does anyone in your household receive a government grant OR income from the government such as…….read out each option and tick yes or no.  IF YES ask:  How many of each type of grant is received (i.e. how many people receive each?) | **Type of grant** | | | **Yes** | **No** | | **If yes, number received** | |
| --- | --- | --- | --- | --- | --- | --- | --- | --- |
|  | Unemployment insurance (UIF) | | | 1 | 0 | |  | |
|  | Worker’s compensation | | | 1 | 0 | |  | |
|  | State old age pension | | | 1 | 0 | |  | |
|  | Disability grant | | | 1 | 0 | |  | |
|  | Child support grant | | | 1 | 0 | |  | |
|  | Care dependency grant | | | 1 | 0 | |  | |
|  | Foster care grant | | | 1 | 0 | |  | |
|  | Grant in aid | | | 1 | 0 | |  | |
|  | Social relief | | | 1 | 0 | |  | |
|  | Other | | | 1 | 0 | |  | |
|  | Don’t know | | | | 99 | |  | |
| **1.17**  If someone in the household receives a disability grant, ask:  Is it you that receives the disability grant? | | | | | Yes | | | 1  **If no go to**  **1.19** |
|  |  |  |  |  | No | | | 0 |
| **1.18**  If YES ask:  What is the reason that you receive this disability grant? | |  | | | | | | |
| **1.19**  If NO ask:  Have you applied for a disability grant? | | | | | Yes | | | 1 |
|  |  |  |  |  | No | | | 0 |
| **1.20**  Where were you born? READ OUT I know this is a sensitive question to ask at this stage, but we are asking because we want to see if health services treat South Africans differently to those who are not from South Africa. | | | South Africa | | | | | 1  **If other go to 1.22** |
|  |  |  | Other (specify) | | | | | |
| **1.21**  If respondent born in South Africa, ask:  Which province were you born in?  Use current province borders | | | Western Cape | | | | | 1 |
|  |  |  | Eastern Cape | | | | | 2 |
|  |  |  | Northern Cape | | | | | 3 |
|  |  |  | Free State | | | | | 4 |
|  |  |  | KwaZulu-Natal | | | | | 5 |
|  |  |  | North West | | | | | 6 |
|  |  |  | Gauteng | | | | | 7 |
|  |  |  | Mpumalanga | | | | | 8 |
|  |  |  | Limpopo | | | | | 9 |
|  |  |  | Don’t Know | | | | | 99 |
| **1.22**  If respondent not born in South Africa, ask:  Do you have a South African ID document? | | | | | | Yes | | 1 |
|  |  |  |  |  |  | No | | 0 |
| **1.23**  Are you covered by a Medical Aid or any scheme that helps you pay for health-care services or medicines? | | | | | | Yes | | 1 |
|  |  |  |  |  |  | No | | 0 |

| **SECTION 2: UTILISATION OF HIV AND OTHER HEALTH SERVICES AND INDIRECT COSTS OF THE DISEASE** | | | | | | |
| --- | --- | --- | --- | --- | --- | --- |
| **READ OUT:** In this section we are asking you some questions about what health care you have used for your HIV. | | | | | | |
| **2.1**  When did you find out you were HIV positive? | | | ___________________________________  MM YYYY | | | |
| **2.2**  When did you FIRST begin receiving antiretroviral (ARV) treatment? | | | ___________________________________  MM YYYY | | | |
| **2.3**  Where were you diagnosed with HIV? | _____________________________________________  Facility name/mobile clinic/at home  _____________________________________________  Province/city/village/township | | | | | |
| **2.4**  How often do you collect your ARV treatment here at the clinic? | | | Monthly or less (weekly/bi-weekly) | | | 1 |
|  |  |  | 2-monthly | | | 2 |
|  |  |  | More than 2 monthly | | | 3 |
| **2.5**  Who, if anyone, supports you in taking your ARV treatment each day? indicate relationship e.g. sister, not name | | | _______________________________  Relationship | | | |
| **2.6**  Have you received ARV treatment from a clinic other than this one? | | | | | Yes | 1 |
|  |  |  |  |  | No | 0  **If yes go to 2.9** |
| **2.7**  Besides ARVs, are you able to get the other health services you need in this facility? | | | | | Yes | 1 |
|  |  |  |  |  | No | 0 |
| **2.8**  If NO ask:  Which services do you have to get elsewhere? | |  | | | | |
| **READ OUT:** Some people find it quite hard to stick to the ARV treatment and might not always be able to make their appointments at the clinic. We are now going to ask you about whether you have had any of these sorts of problems and what the reasons might be. | | | | | | |
| **2.9**  Did you miss taking any of your ARV tablets YESTERDAY? | | | | Yes | | 1 |
|  |  |  |  | No | | 0 |
| **2.10**  Did you miss taking any ARV tablets the day before YESTERDAY? | | | | Yes | | 1 |
|  |  |  |  | No | | 0 |
| **2.11**  Did you miss taking any ARV tablets 3 DAYS AGO? Specify the calendar day in relation to the day of the interview | | | | Yes | | 1 |
|  |  |  |  | No | | 0 |
| **2.12**  Apart from the last three days, have you ever missed taking any ARV tablets? | | | | Yes | | 1 |
|  |  |  |  | No | | 0 |
| **2.13**  Have you missed any visits to the ARV clinic in the last 6 months? | | | | Yes | | 1  **If no go to 2.16** |
|  |  |  |  | No | | 0 |
| **2.14**  IF YES  How many visits did you miss? | | | |  | | |

| **2.15**  What was the reason(s) for missing the visits?  Do not read the list aloud; probe respondent to give you up to three reasons  Tick up to three yes options and tick all others no | | **Reason** | | | | | **Yes** | | | **No** | |
| --- | --- | --- | --- | --- | --- | --- | --- | --- | --- | --- | --- |
|  |  | Lack of money | | | | | 1 | | | 0 | |
|  |  | Lack of time | | | | | 1 | | | 0 | |
|  |  | I felt better | | | | | 1 | | | 0 | |
|  |  | I could not take time off from work | | | | | 1 | | | 0 | |
|  |  | No transport | | | | | 1 | | | 0 | |
|  |  | Too ill to travel | | | | | 1 | | | 0 | |
|  |  | Other responsibilities | | | | | 1 | | | 0 | |
|  |  | The treatment is not effective / does not make me feel better | | | | | 1 | | | 0 | |
|  |  | The queues in the facility are too long | | | | | 1 | | | 0 | |
|  |  | The staff are rude or uncaring | | | | | 1 | | | 0 | |
|  |  | I have had bad experiences with staff in the past | | | | | 1 | | | 0 | |
|  |  | Don’t know | | | | | | | 99 | | |
|  |  | Other 1 (specify) | | | | | | | | | |
|  |  | Other 2 (specify) | | | | | | | | | |
| **2.16**  Apart from visits to this clinic for your ARVs, have you used this clinic or any other health service in the last four weeks?  Specify in relation to the calendar  Read out each option one at a time. IF YES ask:  How many visits (or inpatient days) did you have?  Then ask:  How much did you have to pay the provider for each?  Tick all that apply “Yes” and others “No” | **Type of facility or service** | | | **Yes** | **No** | **If yes, times used** | | **If yes, amount spent** | | |  |
|  | Chemist/pharmacy | | | 1 | 0 |  | |  | | |  |
|  | This clinic (not for ARVs) | | | 1 | 0 |  | |  | | |  |
|  | A different public clinic | | | 1 | 0 |  | |  | | |  |
|  | A private doctor | | | 1 | 0 |  | |  | | |  |
|  | A traditional healer | | | 1 | 0 |  | |  | | |  |
|  | A public hospital emergency/ outpatient department | | | 1 | 0 |  | |  | | |  |
|  | Inpatient stay in a public hospital | | | 1 | 0 |  | |  | | |  |
|  | A private hospital emergency/ outpatient department | | | 1 | 0 |  | |  | | |  |
|  | Inpatient stay in a private hospital | | | 1 | 0 |  | |  | | |  |
|  | TB clinic | | | 1 | 0 | **Leave blank** | | | | |  |
|  | Antenatal clinic [women only] | | | 1 | 0 |  |  |  |  |  |  |
|  | Other (Specify) | | | | | | | | | |  |
| **2.17**  Have you spent any other money on health care in the past month (e.g. traditional medicines, spaza shops, special food, etc). IF YES, how much have you spent? | | | Yes | | | | | | | 1 |  |
|  |  |  | No | | | | | | | 0 |  |
|  |  |  | If Yes, specify amount  _______________________________(Rand) | | | | | | | |  |

| **SECTION 3: AFFORDABILITY** | | | | | | | | | | | | | | | | | | | | | | |
| --- | --- | --- | --- | --- | --- | --- | --- | --- | --- | --- | --- | --- | --- | --- | --- | --- | --- | --- | --- | --- | --- | --- |
| **READ OUT**: I am now going to ask you some questions about the financial difficulties you might face in seeking health care for your HIV/AIDS. | | | | | | | | | | | | | | | | | | | | | | |
| **3.1**  In the last month did you have to borrow money to pay for healthcare? | | | | | | | | | | | | | | Yes | | | | | | | 1  **If no go to 3.3** | |
|  |  |  |  |  |  |  |  |  |  |  |  |  |  | No | | | | | | | 0 | |
| **3.2**  If YES  How much money did you borrow? | | | | | | | | | _____________________________(Rand) | | | | | | | | | | | | | |
| 3.3  In the last month did you have to sell personal or household items in order to pay for healthcare? | | | | | | | | | | | | | Yes | | | | | | | | 1 | |
|  |  |  |  |  |  |  |  |  |  |  |  |  | No | | | | | | | | 0 | |
| **3.4**  How much time did you spend at the clinic last time you came to collect your ARV treatment? | | | | | | | | | ___________ hrs _____________ minutes | | | | | | | | | | | | | |
| **3.5**  How much time did you spend at the clinic last time you came to see the doctor/nurse for your ARVS? | | | | | | | | | ___________ hrs _____________ minutes | | | | | | | | | | | | | |
| **3.6**  What would you have been doing if you weren’t at the clinic today?  Tick all that apply “Yes” and others “No” | | **Activity** | | | | | | | | | | | | | | | | **Yes** | | | | **No** |
|  |  | Working | | | | | | | | | | | | | | | | 1 | | | | 0 |
|  |  | Doing unpaid community work or volunteer work | | | | | | | | | | | | | | | | 1 | | | | 0 |
|  |  | Doing household chores such as cleaning, cooking, shopping for food, maintenance and repairs, working in the garden, gathering wood, gathering water, housework etc. | | | | | | | | | | | | | | | | 1 | | | | 0 |
|  |  | Taking care of children | | | | | | | | | | | | | | | | 1 | | | | 0 |
|  |  | Leisure activities (sport, watching TV, listening to music, reading, visiting friends and family, going to movies etc) | | | | | | | | | | | | | | | | 1 | | | | 0 |
|  |  | Attending school or other educational institution | | | | | | | | | | | | | | | | 1 | | | | 0 |
|  |  | Nothing | | | | | | | | | | | | | | | | 1 | | | | 0 |
|  |  | I don’t know | | | | | | | | | | | | | | | | | | 99 | | |
|  |  | Other (specify) | | | | | | | | | | | | | | | | | | | | |
| **3.7**  In coming to receive treatment today, how much did you pay for:  Read out each item. If no money spent, code as “0” for each item | | **Category** | | | | | | | | | | | | | | **Rand** | | | | | | |
|  |  | Transport (one way) | | | | | | | | | | | | | |  | | | | | | |
|  |  | Clinic/hospital fees | | | | | | | | | | | | | |  | | | | | | |
|  |  | Medicines | | | | | | | | | | | | | |  | | | | | | |
|  |  | Someone to take over your tasks while you are here including childcare | | | | | | | | | | | | | |  | | | | | | |
|  |  | Accommodation if you need to stay the night nearby | | | | | | | | | | | | | |  | | | | | | |
|  |  | Food during visit | | | | | | | | | | | | | |  | | | | | | |
|  |  | Phoning or sms’ing | | | | | | | | | | | | | |  | | | | | | |
|  |  | Other, specify: | | | | | | | | | | | | | |  | | | | | | |
| **3.8**  Did you find it easy or difficult to incur these expenses? Refer to expenses in 3.7 | | | | | | | | | | | | Easy | | | | | | | | | 1 | |
|  |  |  |  |  |  |  |  |  |  |  |  | Difficult | | | | | | | | | 2 | |
|  |  |  |  |  |  |  |  |  |  |  |  | Neither easy nor difficult | | | | | | | | | 3 | |
|  |  |  |  |  |  |  |  |  |  |  |  | Don’t know | | | | | | | | | 99 | |
| **3.9**  If respondent is working  Did you lose income from the time you took from your job to come here today? | | | | | | | | | | | | | | | Yes | | | | | | 1 | |
|  |  |  |  |  |  |  |  |  |  |  |  |  |  |  | No | | | | | | 0  **If no go to 3.11** | |
| **3.10**  If YES:  How much money did you lose? | | | | | | | | | | | |  | | | | | | | | | | |
| **3.11**  Who, if anyone, has been helping you financially, i.e. with cash, buying food, providing transport etc, with your HIV/AIDS?  Tick all that apply “Yes” and others “No” | | | | | | **Person** | | | | | | | | | | | | **Yes** | | | | **No** |
|  |  |  |  |  |  | Husband/wife | | | | | | | | | | | | 1 | | | | 0 |
|  |  |  |  |  |  | Father/mother | | | | | | | | | | | | 1 | | | | 0 |
|  |  |  |  |  |  | Boyfriend/girlfriend | | | | | | | | | | | | 1 | | | | 0 |
|  |  |  |  |  |  | Other relatives | | | | | | | | | | | | 1 | | | | 0 |
|  |  |  |  |  |  | Friends | | | | | | | | | | | | 1 | | | | 0 |
|  |  |  |  |  |  | Nobody | | | | | | | | | | | | 1 | | | | 0 |
|  |  |  |  |  |  | Employer (over and above normal wages) | | | | | | | | | | | | 1 | | | | 0 |
|  |  |  |  |  |  | Don’t know | | | | | | | | | | | | | | 99 | | |
|  |  |  |  |  |  | Other (specify) | | | | | | | | | | | | | | | | |
| **SECTION 4: AVAILABILITY** | | | | | | | | | | | | | | | | | | | | | | |
| **4.1**  Is this the closest clinic to your home that offers ARV treatment? | | | | | | | | | | | Yes | | | | | | | | | | 1  **If yes go to 4.3** | |
|  |  |  |  |  |  |  |  |  |  |  | No | | | | | | | | | | 0 | |
| **4.2**  If NO  Why do you prefer this facility? | | | | |  | | | | | | | | | | | | | | | | | |
| **4.3**  Are the opening hours of this clinic convenient for you? | | | | | | | | Yes | | | | | | | | | | | | | 1 | |
|  |  |  |  |  |  |  |  | No | | | | | | | | | | | | | 0 | |
|  |  |  |  |  |  |  |  | Don’t know | | | | | | | | | | | | | 99 | |
| **4.4**  How did you get here today?  Tick all that apply “Yes” and all others “No” | | | | **Transport mode** | | | | | | | | | | | | | | **Yes** | | | | **No** |
|  |  |  |  | By foot | | | | | | | | | | | | | | 1 | | | | 0 |
|  |  |  |  | Bicycle | | | | | | | | | | | | | | 1 | | | | 0 |
|  |  |  |  | Taxi (can be meter taxi or minibus) | | | | | | | | | | | | | | 1 | | | | 0 |
|  |  |  |  | Bus / Train | | | | | | | | | | | | | | 1 | | | | 0 |
|  |  |  |  | Private car (can be own car, hired, catching a lift) | | | | | | | | | | | | | | 1 | | | | 0 |
|  |  |  |  | Ambulance / hospital transport | | | | | | | | | | | | | | 1 | | | | 0 |
|  |  |  |  | Other (specify) | | | | | | | | | | | | | | | | | | |
| **4.5**  How long did it take you to get here? (one way only) time taken from leaving home to arriving at facility | | | | | | | ___________ hrs _____________ minutes  **If yes go to 4.8** | | | | | | | | | | | | | | | |
| **4.6**  Do you currently belong to a support group | | | | | | | | | | | | Yes | | | | | | | | | 1 | |
|  |  |  |  |  |  |  |  |  |  |  |  | No | | | | | | | | | 0 | |
| **4.7**  If NO  Have you ever belonged to a support group in the past? | | | | | | | | | | | | Yes | | | | | | | | | 1 | |
|  |  |  |  |  |  |  |  |  |  |  |  | No | | | | | | | | | 0 | |
| **4.8**  Do you have a treatment buddy? | | | | | | | | | | | | Yes | | | | | | | | | 1 | |
|  |  |  |  |  |  |  |  |  |  |  |  | No | | | | | | | | | 0 | |
| **4.9**  Do you have a pillbox [show] for keeping your tablets? | | | | | | | | | | | | Yes | | | | | | | | | 1 | |
|  |  |  |  |  |  |  |  |  |  |  |  | No | | | | | | | | | 0 | |
| **4.10**  Since you learnt about your HIV status, has anyone from the health service ever visited you at home for your HIV? | | | | | | | | | | | | Yes | | | | | | | | | 1 | |
|  |  |  |  |  |  |  |  |  |  |  |  | No | | | | | | | | | 0 | |
| **4.11**  Are you able to give me the result of your latest CD4? | | | | | | | | | | | | Yes | | | | | | | | | 1  **If no go to 4.13** | |
|  |  |  |  |  |  |  |  |  |  |  |  | No | | | | | | | | | 0 | |
| **4.12**  If YES write result | | | | | | | CD4 count result | | | | | | | | | | | | | | | |
| **READ OUT** Please tell me if you think the following two statements are true/correct or false/incorrect: | | | | | | | | | | | | | | | | | | | | | | |
| **4.13**  It is acceptable to stop ARVs after gaining weight | | | | | | | | | | | | True / correct | | | | | | | | | 1 | |
|  |  |  |  |  |  |  |  |  |  |  |  | False / incorrect | | | | | | | | | 2 | |
|  |  |  |  |  |  |  |  |  |  |  |  | Don’t know | | | | | | | | | 99 | |
| **4.14**  ARVs cure HIV/AIDS | | | | | | | | | | | | True / correct | | | | | | | | | 1 | |
|  |  |  |  |  |  |  |  |  |  |  |  | False / incorrect | | | | | | | | | 2 | |
|  |  |  |  |  |  |  |  |  |  |  |  | Don’t know | | | | | | | | | 99 | |
| **SECTION 5: ACCEPTABILITY** | | | | | | | | | | | | | | | | | | | | | | |
| **5.1**  Have you told anyone besides the health care workers that you are HIV positive? | | | | | | | | | | | | Yes | | | | | | | | | 1  **If no go** **to 5.3** | |
|  |  |  |  |  |  |  |  |  |  |  |  | No | | | | | | | | | 0 | |
| **5.2**  IF YES  Who have you told about your HIV status? indicate relationship e.g. sister, not name | | | | | | | | |  | | | | | | | | | | | | | |
| **5.3**  To what extent do you agree with the following statement:  “I have all the support from my partner that I need to cope with my illness”? | | | | | | | | | | | | Agree | | | | | | | | 1 | | |
|  |  |  |  |  |  |  |  |  |  |  |  | Disagree | | | | | | | | 2 | | |
|  |  |  |  |  |  |  |  |  |  |  |  | Don’t know | | | | | | | | 99 | | |
|  |  |  |  |  |  |  |  |  |  |  |  | Not applicable | | | | | | | | 98 | | |
| **5.4**  “I have all the support that I need from my family” | | | | | | | | | | | | Agree | | | | | | | | 1 | | |
|  |  |  |  |  |  |  |  |  |  |  |  | Disagree | | | | | | | | 2 | | |
|  |  |  |  |  |  |  |  |  |  |  |  | Don’t know | | | | | | | | 99 | | |
|  |  |  |  |  |  |  |  |  |  |  |  | Not applicable | | | | | | | | 98 | | |
| **5.5**  “I have all the support that I need from my friends” | | | | | | | | | | | | Agree | | | | | | | | 1 | | |
|  |  |  |  |  |  |  |  |  |  |  |  | Disagree | | | | | | | | 2 | | |
|  |  |  |  |  |  |  |  |  |  |  |  | Don’t know | | | | | | | | 99 | | |
|  |  |  |  |  |  |  |  |  |  |  |  | Not applicable | | | | | | | | 98 | | |
| **5.6**  Do you feel that people in the community judge you negatively for attending this facility for your ARV treatment? | | | | | | | | | | | | Yes | | | | | | | | 1 | | |
|  |  |  |  |  |  |  |  |  |  |  |  | No | | | | | | | | 0 | | |
|  |  |  |  |  |  |  |  |  |  |  |  | Don’t know | | | | | | | | 99 | | |
| **5.7**  For your ARV treatment what would you prefer:   1. To see a nurse in a nearby clinic or 2. To travel further to see a doctor | | | | | | | | | | | | Nurse | | | | | | | | 1 | | |
|  |  |  |  |  |  |  |  |  |  |  |  | Doctor | | | | | | | | 2 | | |
|  |  |  |  |  |  |  |  |  |  |  |  | Indifferent | | | | | | | | 3 | | |
|  |  |  |  |  |  |  |  |  |  |  |  | Don’t know | | | | | | | | 99 | | |
| **5.8**  In this clinic are you able to talk to the doctors or nurses in private? | | | | | | | | | | | | Always | | | | | | | | 1 | | |
|  |  |  |  |  |  |  |  |  |  |  |  | Sometimes | | | | | | | | 2 | | |
|  |  |  |  |  |  |  |  |  |  |  |  | Never | | | | | | | | 3 | | |
| **READ OUT:** Can you tell me whether you agree or disagree with these statements when thinking about your general experience in this clinic? | | | | | | | | | | | | | | | | | | | | | | |
| **5.9**  The queues to see a doctor or nurse are too long at this facility | | | | | | | | | | Agree | | | | | | | | | | | 1 | |
|  |  |  |  |  |  |  |  |  |  | Disagree | | | | | | | | | | | 0 | |
|  |  |  |  |  |  |  |  |  |  | Both agree and disagree | | | | | | | | | | | 2 | |
|  |  |  |  |  |  |  |  |  |  | Don’t know / not sure | | | | | | | | | | | 99 | |
| **5.10**  The doctors and nurses (*health workers)* discussed the treatment fully with me | | | | | | | | | | Agree | | | | | | | | | | | 1 | |
|  |  |  |  |  |  |  |  |  |  | Disagree | | | | | | | | | | | 0 | |
|  |  |  |  |  |  |  |  |  |  | Both agree and disagree | | | | | | | | | | | 2 | |
|  |  |  |  |  |  |  |  |  |  | Don’t know / not sure | | | | | | | | | | | 99 | |
| **5.11**  It is a problem that the *health workers* DO NOT speak my language. | | | | | | | | | | Agree | | | | | | | | | | | 1 | |
|  |  |  |  |  |  |  |  |  |  | Disagree | | | | | | | | | | | 0 | |
|  |  |  |  |  |  |  |  |  |  | Both agree and disagree | | | | | | | | | | | 2 | |
|  |  |  |  |  |  |  |  |  |  | Don’t know / not sure | | | | | | | | | | | 99 | |
| **5.12**  I find it easy to tell the *health workers* when I have missed taking my tablets | | | | | | | | | | Agree | | | | | | | | | | | 1 | |
|  |  |  |  |  |  |  |  |  |  | Disagree | | | | | | | | | | | 0 | |
|  |  |  |  |  |  |  |  |  |  | Both agree and disagree | | | | | | | | | | | 2 | |
|  |  |  |  |  |  |  |  |  |  | Don’t know / not sure | | | | | | | | | | | 99 | |
| **5.13**  The *health workers* are too busy to listen to my problems | | | | | | | | | | Agree | | | | | | | | | | | 1 | |
|  |  |  |  |  |  |  |  |  |  | Disagree | | | | | | | | | | | 0 | |
|  |  |  |  |  |  |  |  |  |  | Both agree and disagree | | | | | | | | | | | 2 | |
|  |  |  |  |  |  |  |  |  |  | Don’t know / not sure | | | | | | | | | | | 99 | |
| **5.14**  Patient information is kept confidential in this clinic | | | | | | | | | | Agree | | | | | | | | | | | 1 | |
|  |  |  |  |  |  |  |  |  |  | Disagree | | | | | | | | | | | 0 | |
|  |  |  |  |  |  |  |  |  |  | Both agree and disagree | | | | | | | | | | | 2 | |
|  |  |  |  |  |  |  |  |  |  | Don’t know / not sure | | | | | | | | | | | 99 | |
| **5.15**  Some staff DO NOT treat patients with sufficient respect | | | | | | | | | | Agree | | | | | | | | | | | 1 | |
|  |  |  |  |  |  |  |  |  |  | Disagree | | | | | | | | | | | 0 | |
|  |  |  |  |  |  |  |  |  |  | Both agree and disagree | | | | | | | | | | | 2 | |
|  |  |  |  |  |  |  |  |  |  | Don’t know / not sure | | | | | | | | | | | 99 | |
| **5.16**  The health workers I see respect me | | | | | | | | | | Agree | | | | | | | | | | | 1 | |
|  |  |  |  |  |  |  |  |  |  | Disagree | | | | | | | | | | | 0 | |
|  |  |  |  |  |  |  |  |  |  | Both agree and disagree | | | | | | | | | | | 2 | |
|  |  |  |  |  |  |  |  |  |  | Don’t know / not sure | | | | | | | | | | | 99 | |
| **5.17**  The facilities (including waiting area and toilets) are dirty | | | | | | | | | | Agree | | | | | | | | | | | 1 | |
|  |  |  |  |  |  |  |  |  |  | Disagree | | | | | | | | | | | 0 | |
|  |  |  |  |  |  |  |  |  |  | Both agree and disagree | | | | | | | | | | | 2 | |
|  |  |  |  |  |  |  |  |  |  | Don’t know / not sure | | | | | | | | | | | 99 | |
| **5.18**  How satisfied were you with the service today? | | | | | | | | | | Very satisfied/ Satisfied | | | | | | | | | | | 1 | |
|  |  |  |  |  |  |  |  |  |  | Neither satisfied nor dissatisfied | | | | | | | | | | | 2 | |
|  |  |  |  |  |  |  |  |  |  | Dissatisfied/ Very dissatisfied | | | | | | | | | | | 3 | |
|  |  |  |  |  |  |  |  |  |  | Don’t know | | | | | | | | | | | 99 | |
| **5.19**  Since you first started coming to this facility, have you ever left without being helped? | | | | | | | | | | Yes | | | | | | | | | | | 1  **If no go to 5.21** | |
|  |  |  |  |  |  |  |  |  |  | No | | | | | | | | | | | 0 | |
| **5.20**  IF YES  Can you explain what happened? | | |  | | | | | | | | | | | | | | | | | | | |
| **5.21**  Have you ever not used ARV services when you needed them? | | | | | | | | | | Yes | | | | | | | | | | | 1  **If no go to 5.21** | |
|  |  |  |  |  |  |  |  |  |  | No | | | | | | | | | | | 0 | |
| **5.22**  IF YES  Why did you not use ARV services?  Include all factors – personal and facility-related | | |  | | | | | | | | | | | | | | | | | | | |
| **5.23**  How do you think the service in this clinic could be improved?  Tick all that apply “Yes” and all others “No” | | | **Improvement** | | | | | | | | | | | | | | **Yes** | | | | | **No** |
|  |  |  | Shorter queues | | | | | | | | | | | | | | 1 | | | | | 0 |
|  |  |  | More health workers | | | | | | | | | | | | | | 1 | | | | | 0 |
|  |  |  | Cleaner facilities | | | | | | | | | | | | | | 1 | | | | | 0 |
|  |  |  | Better patient facilities (toilets, waiting room area etc) | | | | | | | | | | | | | | 1 | | | | | 0 |
|  |  |  | Don’t know | | | | | | | | | | | | | | | | 99 | | | |
|  |  |  | Other (specify) | | | | | | | | | | | | | | | | | | | |
| **SECTION 6: DWELLING CHARACTERISTICS, HOUSEHOLD INCOME, EXPENDITURE AND HOUSEHOLD ASSETS** | | | | | | | | | | | | | | | | | | | | | | |
| READ OUT Finally, we want to ask you some questions about the characteristics of the house where you live and type of facilities available within your household | | | | | | | | | | | | | | | | | | | | | | |
| **6.1**  Where do you live? | ______________________________________________village or community  _________________________________________________area or township | | | | | | | | | | | | | | | | | | | | | |
| **6.2**  Which best describes the type of house in which you live?  Clarify answer  Tick one only | House or brick structure on a separate stand or yard or on farm | | | | | | | | | | | | | | | | | | | | 1 | |
|  | Traditional dwelling/hut/structure made of traditional materials | | | | | | | | | | | | | | | | | | | | 2 | |
|  | Flat or apartment in block of flats | | | | | | | | | | | | | | | | | | | | 3 | |
|  | Town/cluster/semi-detached house (simplex, duplex or triplex) | | | | | | | | | | | | | | | | | | | | 4 | |
|  | Unit in retirement village | | | | | | | | | | | | | | | | | | | | 5 | |
|  | Dwelling/house/flat/room in backyard | | | | | | | | | | | | | | | | | | | | 6 | |
|  | Informal dwelling/shack IN the backyard of a formal house | | | | | | | | | | | | | | | | | | | | 7 | |
|  | Informal dwelling/shack NOT in backyard e.g. in an informal/squatter settlement or on farm | | | | | | | | | | | | | | | | | | | | 8 | |
|  | Room/flatlet not in backyard but on a shared property e.g granny flat | | | | | | | | | | | | | | | | | | | | 9 | |
|  | Caravan/tent | | | | | | | | | | | | | | | | | | | | 10 | |
|  | Worker’s hostel | | | | | | | | | | | | | | | | | | | | 11 | |
|  | Other (specify) | | | | | | | | | | | | | | | | | | | | | |

| **6.3**  What is the main material of your house’s walls?  Clarify answer  Tick one only | | Bricks & plaster/finished | | 1 |
| --- | --- | --- | --- | --- |
|  |  | Bare brick/cement block | | 2 |
|  |  | Corrugated iron/zinc | | 3 |
|  |  | Wood | | 4 |
|  |  | Plastic | | 5 |
|  |  | Cardboard | | 6 |
|  |  | Mixture of mud and cement | | 7 |
|  |  | Wattle and daub | | 8 |
|  |  | Mud | | 9 |
|  |  | Other (specify) | | |
| **6.4**  What is the main material of your house’s roof?  Clarify answer  Tick one only | | Tiles | | 1 |
|  |  | Corrugated iron/zinc | | 2 |
|  |  | Thatching | | 3 |
|  |  | Asbestos | | 4 |
|  |  | Plastic | | 5 |
|  |  | Cardboard | | 6 |
|  |  | Other (specify) | | |
| **6.5**  How many rooms, including kitchens, does your house have? Interviewer, probe and exclude bathrooms, sheds, garages, stables, etc. from the total unless people are living in them | | |  | |
| **6.6**  What is the main source of **drinking water** for members of your household?  Clarify answer  Tick one only | Piped (tap) water in dwelling | | | 1 |
|  | Piped (tap) water on site or in yard | | | 2 |
|  | Borehole on site | | | 3 |
|  | Rain water tank on site | | | 4 |
|  | Neighbour’s tap | | | 5 |
|  | Public/communal tap (either free or paid) | | | 6 |
|  | Water carrier/tanker | | | 7 |
|  | Borehole off site/communal | | | 8 |
|  | Flowing water/stream/river | | | 9 |
|  | Stagnant water/dam/pool | | | 10 |
|  | Well | | | 11 |
|  | Spring | | | 12 |
|  | Other (specify) | | | |

| **6.7**  What type of toilet does your household use?  Clarify answer  Tick one only | | Flush toilet (connected to sewage) | | | 1 |
| --- | --- | --- | --- | --- | --- |
|  |  | Flush toilet (with septic tank) | | | 2 |
|  |  | Chemical toilet | | | 3 |
|  |  | Pit latrine with ventilation pipe | | | 4 |
|  |  | Pit latrine without ventilation pipe | | | 5 |
|  |  | Bucket toilet | | | 6 |
|  |  | No facility/bush/field | | | 7 |
|  |  | Other (specify) | | | |
| **6.8**  What is the main source of energy for cooking in your household?  Clarify answer  Tick one only | | Electricity from mains | | | 1 |
|  |  | Electricity from generator | | | 2 |
|  |  | Gas | | | 3 |
|  |  | Paraffin | | | 4 |
|  |  | Wood | | | 5 |
|  |  | Coal | | | 6 |
|  |  | Animal dung | | | 7 |
|  |  | Solar energy | | | 8 |
|  |  | Other (specify) | | | |
| **6.9**  Does your household have any of the following items in good working order?  Read out each item and tick all that apply “Yes” and all others “No” | |  | **Yes** | | **No** |
|  |  | Telkom / landline phone | 1 | | 0 |
|  |  | Cell phone | 1 | | 0 |
|  |  | Radio | 1 | | 0 |
|  |  | Television | 1 | | 0 |
|  |  | Video recorder/DVD player | 1 | | 0 |
|  |  | Electric stove with oven | 1 | | 0 |
|  |  | Bicycle | 1 | | 0 |
|  |  | Personal computer at home | 1 | | 0 |
|  |  | Internet facilities at home | 1 | | 0 |
|  |  | Fridge | 1 | | 0 |
|  |  | Car/truck/bakkie | 1 | | 0  **If no go to 6.16** |
| **6.10**  Does your household own cattle, livestock or chickens? | | | Yes | | 1 |
|  |  |  | No | | 0 |
| **6.11**  IF YES  How many cattle does the household own? | | __________________________(Number of cattle) | | | |
|  |  | None | | 0 | |
|  |  | Don’t know | | 99 | |
| **6.12**  IF YES  How many goats does the household own? | | __________________________(Number of goats) | | | |
|  |  | None | | 0 | |
|  |  | Don’t know | | 99 | |
| **6.13**  IF YES  How many chickens does the household own? | | ________________________(Number of chickens) | | | |
|  |  | None | | 0 | |
|  |  | Don’t know | | 99 | |
| **6.14**  IF YES  How many pigs does the household own? | | __________________________(Number of pigs) | | | |
|  |  | None | | 0 | |
|  |  | Don’t know | | 99 | |
| **6.15**  IF YES  Does the HH own any other farm animals? IF YES What are they  How many [other] does the household own? | | (Other, specify) | | | |
|  |  | __________________________(Number of other) | | | |
|  |  | None | | 0 | |
|  |  | Don’t know | | 99 | |
| **6.16**  In general how much does your household usually spend in a month?  If the respondent does not give you a precise estimate ask him/her  In which of the following ranges, would you say your household EXPENDITURE generally falls?  Tick one only | | ________________________________Rand | | | |
|  |  | R0 – R399 | | 1 | |
|  |  | R400 – R799 | | 2 | |
|  |  | R800 – R1 199 | | 3 | |
|  |  | R1 200 - R1 799 | | 4 | |
|  |  | R1 800 - R2 499 | | 5 | |
|  |  | R2 500 - R4 999 | | 6 | |
|  |  | R5 000 - R9 999 | | 7 | |
|  |  | R10 000 or more | | 8 | |
|  |  | Don’t know | | 99 | |
|  |  | Refuse | | 97 | |
| **6.17**  Do you have anything else that you would like to tell us about your experience of seeking or receiving care at this facility? |  | | | | |
| **6.18**  Note the end time of the interview | : am / pm | | | | |

**Thank the interviewee and indicate that you would now like to ask his/her permission to examine his/her medical record**
